# Methylglyoxal-Modified Albumin Effects on Endothelial Arginase Enzyme and Vascular Function

**DOI:** 10.3390/cells12050795

**Published:** 2023-03-03

**Authors:** Ebaa M. Alzayadneh, Alia Shatanawi, R. William Caldwell, Ruth B. Caldwell

**Affiliations:** 1 Department of Physiology and Biochemistry, School of Medicine, University of Jordan, Amman 11942, Jordan; 2 Department of Pharmacology, School of Medicine, University of Jordan, Amman 11942, Jordan; 3 Department of Pharmacology and Toxicology, Augusta University, Augusta, GA 30912, USA; 4 Culver Vision Discovery Institute, Augusta University, Augusta, GA 30912, USA; 5 Department of Cellular Biology and Anatomy, Medical College of Georgia, Augusta University, Augusta, GA 30912, USA; 6 Vascular Biology Center, Augusta University, Augusta, GA 30912, USA

**Keywords:** advanced glycation end products, arginase, endothelial cells, diabetes, vascular dysfunction, nitric oxide

## Abstract

Advanced glycation end products (AGEs) contribute significantly to vascular dysfunction (VD) in diabetes. Decreased nitric oxide (NO) is a hallmark in VD. In endothelial cells, NO is produced by endothelial NO synthase (eNOS) from L-arginine. Arginase competes with NOS for L-arginine to produce urea and ornithine, limiting NO production. Arginase upregulation was reported in hyperglycemia; however, AGEs’ role in arginase regulation is unknown. Here, we investigated the effects of methylglyoxal-modified albumin (MGA) on arginase activity and protein expression in mouse aortic endothelial cells (MAEC) and on vascular function in mice aortas. Exposure of MAEC to MGA increased arginase activity, which was abrogated by MEK/ERK1/2 inhibitor, p38 MAPK inhibitor, and ABH (arginase inhibitor). Immunodetection of arginase revealed MGA-induced protein expression for arginase I. In aortic rings, MGA pretreatment impaired acetylcholine (ACh)-induced vasorelaxation, which was reversed by ABH. Intracellular NO detection by DAF-2DA revealed blunted ACh-induced NO production with MGA treatment that was reversed by ABH. In conclusion, AGEs increase arginase activity probably through the ERK1/2/p38 MAPK pathway due to increased arginase I expression. Furthermore, AGEs impair vascular function that can be reversed by arginase inhibition. Therefore, AGEs may be pivotal in arginase deleterious effects in diabetic VD, providing a novel therapeutic target.

## 1. Introduction

Vascular dysfunction (VD) contributes to several diabetic complications and its pathophysiology is intricately linked to oxidative stress and inflammation. Advanced glycation end products (AGE) and arginase enzyme have been shown separately to play roles in VD; however, the relationship between these two factors in diabetic VD is not yet clear. Arginase is well demonstrated as an important enzyme in urea cycle, detoxifying ammonia by hydrolyzing L-arginine to ornithine and urea. There are two identified isoforms encoded by different genes, arginase I and II; however, they share similar mechanisms and metabolites [1,2]. Arginase is constitutively expressed in human endothelial cells in both isoforms, where arginase I is located in the cytosol, and arginase II in mitochondria of human endothelial cells [3,4]. In addition to its role in the urea cycle, arginase produces ornithine required for polyamines and L-proline synthesis involved in cell proliferation, differentiation, and repair [5]. There is a growing body of evidence indicating that constitutive levels of arginase activity in endothelium limit NO synthesis and NO-dependent vasodilatory function [6,7,8]. Arginase was shown to be induced by various stimuli such as oxidative stress, oxidized lipoproteins, tumor necrosis factor (TNFα), and hypoxia [9,10,11,12,13,14].

Upregulation of arginase was also demonstrated in cells exposed to high glucose and in diabetic animal models. High glucose increased arginase activity and limited NO production in bovine coronary endothelial cells in a Rho-kinase-dependent pathway, in which siRNA knockdown of arginase I prevented high-glucose-induced changes [15]. Arginase upregulation was shown to be mediated by reactive oxygen species (ROS) and the PKC/Rho A pathway [9]. Interestingly, both arginase and endothelial nitric oxide synthase (eNOS) contributed to high-glucose-induced superoxide production, due to uncoupling of eNOS associated with diminished availability of L-arginine [9,16]. The functional impairment associated with increased arginase expression and activity in diabetes was demonstrated in isolated vascular preparations and under in vivo conditions [17]. Both mRNA expression and activity of arginase were increased in aorta and liver of a streptozotocin-induced diabetic rat model [15]. Impaired endothelium-dependent vasorelaxation of coronary arteries from rats with type 1 diabetes was normalized by arginase inhibition [15]. Moreover, aortic and retinal endothelial dysfunction in streptozotocin-induced type 1 diabetes was linked to increased arginase expression [18,19]. The role of arginase for vascular dysfunction in vivo was investigated in type 2 diabetic rats, in which arginase inhibition improved myocardial microvascular dysfunction by increased NO availability [20]. Additionally, arginase has been identified as a key player in skeletal muscle arteriolar endothelial dysfunction in a diabetic rat model, where inhibition of arginase restored flow-induced vasodilation [21]. Arginase upregulation and vasodilation impairment were reported in cavernous tissue of diabetic rats linked to extracellular signal–regulated kinase (ERK1/2) [22]. Clinical studies on diabetic patients supported earlier findings on animal studies indicating a significant role for arginase in endothelial dysfunction. Plasma arginase activity was elevated in patients with type 2 diabetes mellitus in comparison with healthy subjects and correlated positively with fasting plasma glucose levels and glycosylated hemoglobin HbA1c levels [23]. Furthermore, arginase levels in plasma were associated with markers of oxidative stress and HbA1c [23]. Functionally, coronary arterioles obtained from patients with diabetes displayed reduced endothelium-dependent relaxation in vitro and increased expression of arginase I in endothelial cells [24]. The endothelium-dependent vasodilatation of coronary arterioles was enhanced by arginase inhibition [24]. In addition, an in vivo study demonstrated that arginase inhibition markedly improves endothelium-dependent vasodilatation in the forearm of patients with type 2 diabetes and coronary artery disease, while it does not affect endothelial function in healthy controls [25].

On the other hand, AGEs, the products of non-enzymatic glycation and oxidation of proteins and lipids that accumulate in diabetes, together with their signal transduction receptor (RAGE), are linked to both the etiology and pathological consequences of types 1 and 2 diabetes [26,27]. AGEs form to an accelerated degree in hyperglycemia and accumulate in the blood vessel wall, directly modifying proteins by the formation of cross-links primarily in the basement membrane and the extracellular matrix [26,27]. Furthermore, circulating AGEs interact with endothelial RAGEs to transduce multiple signaling pathways, which lead to perturbation of cellular functions [27]. RAGE is a member of the immunoglobulin superfamily that binds to multiple ligands such as AGEs, HMGB-1, S100 proteins, or amyloid beta peptide [28,29,30]. Engagement of RAGE to its agonists activates several pathways that result in activating NADPH oxidases, ROS production, ERK, P38 MAP-kinase, JAK/STAT pathway, phospho-inisitol-3 kinases, and NfκB pathway, which culminate in the upregulation of RAGE and other profibrotic and proinflammatory target genes [27].

Clinically, the levels of serum AGEs in patients with type 2 diabetes are inversely related to the degree of endothelium-dependent and endothelium-independent vasodilation [31]. Several mechanisms by which AGEs affect NO bioavailability were suggested in the literature and mostly relate to eNOS. AGEs may reduce the stability of eNOS or impair NO production via RAGE-induced deactivation of the eNOS enzyme [32,33]. To our knowledge, it is still not clear if AGEs directly affect arginase activity, arginase expression, or NO bioavailability in endothelial cells. Given that AGEs via RAGE induce ROS formation and ERK1/2 activation, which are also signaling pathways implicated in arginase stimulation in diabetic vasculature, as shown previously, we sought to investigate the effect of AGE (MGA) on arginase activity and expression. We hypothesized that AGEs may upregulate arginase enzymes, leading to a reduction in the availability of arginine and NO, thus causing deleterious effects on vascular function.

## 2. Materials and Methods

### 2.1. Cell Culture and Treatments

In all cell experiments, mouse aortic endothelial cells (MAECs) were utilized. Proliferating MAECs were purchased from Cell Applications, San Diego, CA, USA. Cells were cultured in Endothelial Growth Medium (Cell Applications, San Diego, CA, USA) and maintained in a humidified atmosphere at 37 °C and 5% CO_2_. Cells were adapted to grow in M199 supplemented with 50 µM L-arginine (Invitrogen, Carlsbad, CA, USA) for 72 h before the experiment to match the normal plasma L-arginine concentration (40 to 100 µM). In addition, 10% FBS (Catalog # SH30396, hyClone, GE Healthcare Life Sciences South Logan, UT, USA), 1% penicillin/streptomycin, and 1% L-glutamine were added to cell growth medium. Cells used for experiments are from 3 to 9 passage numbers. When cells reached 80% confluency, they were serum-starved overnight in M199 supplemented with 50 µM L-arginine, 1% L-glutamine, 1% penicillin/streptomycin, and 0.2% FBS. Glycated albumin (MGA) was prepared as described and characterized previously [34,35]. Briefly, 500 μM methylglyoxal (Sigma, Catalog #M0252, St. Louis, MO, USA) was incubated with 100 μM BSA (Sigma) dissolved in phosphate-buffered saline (PBS) for 24 h, then washed on 10 kDa filters (Macrosep^®^ Advance Device, Pall Life Sciences, MI, USA) to remove excess methylglyoxal, reconstituted with M199 serum-free media, and passed through a 0.2 μm filter [34,35]. In subsets of cells, the inhibitors for arginase, namely boronic acids 2(S)-amino-6-boronohexanoic acid (ABH) (1 mM, ChemCruz, Catalog #221197, Dallas, TX, USA), p38 MAPK, SB-202190 (10 µM) (EMD biosciences, Catalog #S7076, San Diego, CA, USA), and mitogen-activated protein kinase kinase MEK/ERK1/2, PD98059 (EMD biosciences, Catalog #P215, San Diego, CA, USA) (10 µM), were used and added 2 h before the addition of MGA (100 µM) (Sigma-Aldrich, St. Louis, MO, USA) for 24 h; inhibitor concentrations and durations were as previously described [36]. Independent experiments (3–5) were carried out from different passages.

### 2.2. Arginase Activity

Arginase activity was measured using a colorimetric determination of urea production from L-arginine as described previously [37]. Cells were lysed in Tris buffer (50 mM Tris-HCI, 0.1 mM EDTA and EGTA, pH 7.5) containing protease inhibitors (Catalog # P8340, Sigma, St. Louis, MO, USA). These mixtures were subjected to three freeze–thaw cycles and then centrifuged for 10 min at 20,000× *g*. The supernatants were used for arginase activity assay. In brief, 25 µL of supernatant was heated with MnCl2 (10 mM) for 10 m at 56 °C to activate arginase. The mixture was then incubated with 50 µL L-arginine (0.5 M, pH 9.7) for one hour at 37 °C to hydrolyze the L-arginine. The hydrolysis reaction was stopped with acid and the mixture was then heated at 100 °C with 25 µL of α-isonitrosopropiophenone (9% α-ISPF in EtOH) for 45 min. The samples were kept in the dark at room temperature for 10 min; then, absorbance was measured at 540 nm.

### 2.3. Immunodetection of Arginase

Cells were lysed in RIPA buffer (#ab156034, Abcam, Boston, MA, USA) having protease and phosphatase inhibitors (Catalog #P5726 and P0044, Sigma, St. Louis, MO, USA). Cell lysates were centrifuged for 10 min at 20,000× *g*, and supernatants were collected for Western blotting analysis. Protein estimation was conducted in supernatants using a protein assay kit (Bio Rad, Hercules, CA, USA). Equal amounts of protein were loaded, separated by electrophoresis using 10% SDS-PAGE gels, and transferred into nitrocellulose membranes. The blots were blocked using 5% bovine serum albumin (Sigma, St. Louis, MO, USA), incubated with their respective primary and secondary antibodies, anti-arginase 1 (Santa Cruz, Catalog #166920, 1:1000, Dallas, TX, USA), anti-arginase-2 (Santa Cruz, Catalog #393496, 1:1000, Dallas, TX, USA), anti-GAPDH (Catalog #abx005569, 1:10,000, abbexa, Cambridge, UK), followed by the respective secondary antibodies. Signals were detected using chemiluminescence (PierceTM ECL Western, Thermophisher, IL, USA) and the ChemiDoc MP imaging system (Bio-Rad, Hercules, CA, USA). To quantify the resultant blots, individual band intensities were measured (arbitrary units) and ratios of protein to GAPDH were calculated per sample using NIH ImageJ softwareversion 1.53.

### 2.4. Histochemical Detection of Intracellular NO

For the detection of intracellular NO, endothelial cells (1.2 × 10^5^ cells) were plated on a non-coated cover slide (18 × 18 mm) and starved for 24 h prior to treatment; cells were treated with either bovine serum albumin (100 μM) or MGA (100 μM) for 24 h. For cells with inhibition conditions, inhibitors L-NAME (Abcam, Catalog #120136, 1 mM, UK) or ABH (1 mM) were added 30 min before the addition of incubation media (DAF-2DA, Catalog #ab145283, 5 μM, for 40 min, Abcam, in serum-free media) according to the manufacturer’s instructions and as previously described [38]. To promote NO generation by NOS, subsets of cells were treated with acetylcholine (1 μM, Sigma) and L-arginine (1 mM, Sigma) to intensify the signal during the 40 min incubation. Then, cells were washed with PBS twice and fixed in 2% paraformaldehyde for 3 min at 0 °C, and mounted on a slide with mounting media as reported previously [39]. Cells were directly observed under an inverted fluorescence microscope (AxioObserver.Z1; Zeiss, Jena, Germany). The quantification of fluorescence intensity of representative images from 3 independent experiments was carried out using NIH ImageJ software version 1.53.

### 2.5. Animals

Vascular function experiments were performed on aortas obtained from C57BL/6J wild-type mice aged 10 months. Protocols were approved by the Institutional Animal Care and Use Committee of the Medical College of Georgia (Animal Welfare Assurance no. D16-00197).

### 2.6. Vascular Function

Vascular function was assessed as described previously [40]. Following deep anesthesia, tissues were harvested, and mouse aortas were rapidly excised and placed immediately in ice-cold Krebs–Henseleit buffer (NaCl, 118 mM; NaHCO3, 25 mM; glucose, 5.6 mM; KCl, 4.7 mM; KH2PO4, 1.2 mM; MgSO4 7H2O, 1.17 mM and CaCl2 2H2O, 2.5 mM), cleaned, and cut into 2–3 mm segments. Thereafter, aortic rings were placed in M199 serum-free media supplied with 50 μM L-arginine with or without the addition of MGA and the arginase inhibitor (ABH, 1 mM) for 24 h at 37 °C in culture chambers. Aortic rings (3–4 for each condition) were mounted in an oxygenated wire myograph chamber (Danish Myo Technology, Ann Arbor, MI, USA). Tissues were allowed to equilibrate at a resting tension of 5 mN for 1 h with buffer changes. Following phenylephrine (1 μM) precontraction, relaxation curves were performed using progressive doses of acetylcholine (ACh, endothelium-dependent vasodilator) or sodium nitroprusside (SNP, endothelium-independent vasodilator). Changes in tension were measured by a force transducer. A 1 h equilibration was performed between subsequent relaxation curves. Vasorelaxation responses were calculated as the percentage of phenylephrine-induced contraction.

### 2.7. Statistical Analysis

Data are given as mean ± SEM. For multiple comparisons, statistical analysis was performed by one-way analysis of variance (ANOVA) with the Tukey post test. For single comparisons, statistical differences were determined by the Student T test. Differences in concentration–response curves were determined using two-way repeated measures ANOVA. Independent experiments were performed 3–6 times. All statistical analyses were performed with GraphPad Prism version 8.01 (San Diego, CA, USA). Results were considered significant when *p* < 0.05.

## 3. Results

### 3.1. Arginase Activity

**Figure 1:**
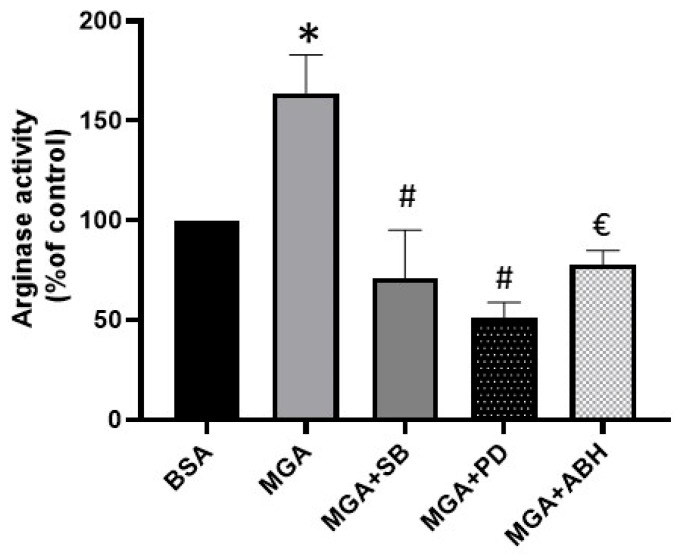
Elevation of arginase activity by exposure of MAEC to MGA (100 µM, 24 h) that was abrogated by pretreatment of cells with SB (10 µM), PD (10 µM), and ABH (1 mM). * *p* < 0.01 control vs. MGA, # *p* ≤ 0.0001 MGA vs. MGA + SB or PD, € *p* < 0.001 MGA vs. MGA + ABH. Values are expressed as means ± SE from 5 independent experiments carried out in triplicates.

Treatment of endothelial cells (MAEC) with (100 μM, 24 h) MGA increased arginase activity by 64% compared to the control BSA-treated cells (*p* < 0.001), as shown in Figure 1. This increase was abrogated when cells were pretreated with the inhibitor of p38 MAPK, SB-202190 (10 µM), or the inhibitor of MEK/ERK1/2, PD98059 (10 µM), or the inhibitor of arginase, ABH (1 mM); n = 5 independent experiments.

### 3.2. Arginase Expression

**Figure 2:**
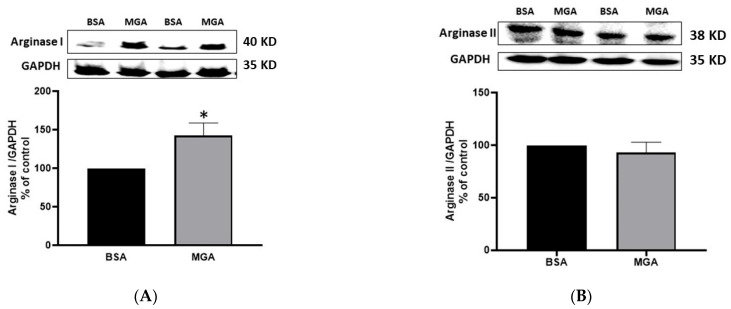
Effect of MGA on arginase expression. (**A**) Immunoblotting analysis of arginase I showing increased expression due to MGA treatment (100 μM, 24 h) as compared to BSA (100 μM, 24 h). (**B**) Immunoblotting analysis of arginase II showing no change in expression after MGA treatment (100 μM, 24 h) as compared to BSA (100 μM, 24 h). Values are expressed as means ± SE from 5 independent experiments carried out in triplicates. * *p* < 0.05 vs. MGA.

MGA treatment (100 μM, 24 h) increased arginase I immunodetected protein expression by 41.6% (*p* < 0.05, n = 5) compared to control BSA conditions, as shown in Figure 2A; however, arginase II expression was not altered, as demonstrated in Figure 2B. These findings indicate that arginase I is the isoform that mainly contributed to the increased arginase activity shown in this study.

### 3.3. Histochemical Detection of Intracellular NO

**Figure 3:**
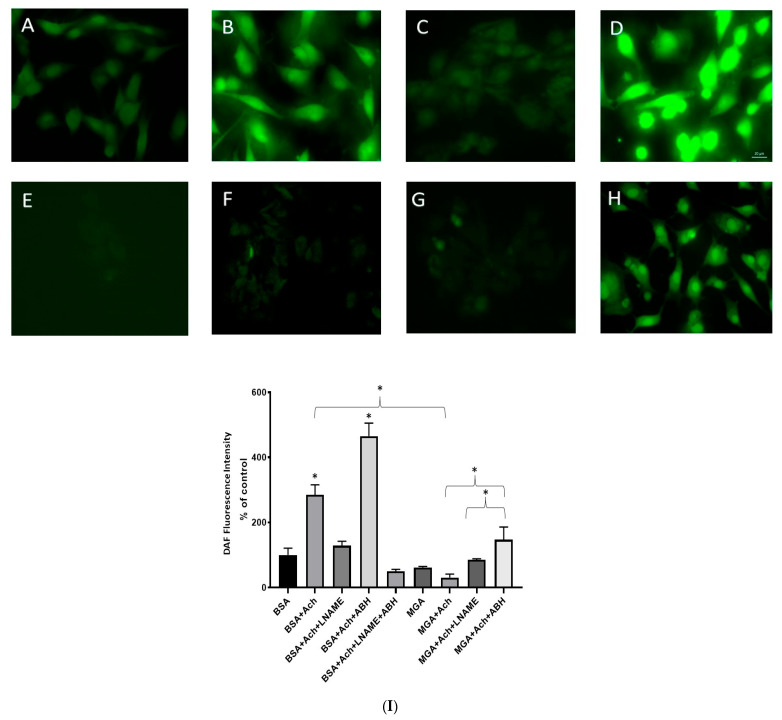
Fluorescent microscopy images of fixed MAECs in 2% formaldehyde after incubation with DAF-2DA (5 μM, 40 min). All cells were pretreated with either BSA (100 µM, 24 h) in (**A**–**D**), or MGA (100 µM, 24 h) in (**E**–**H**). Panels of different cells treatments are as follows: (**A**) BSA without ACh induction, (**B**) BSA with ACh induction, (**C**) BSA with ACh induction and pretreated with L-NAME, (**D**) BSA with ACh induction and pretreated with ABH, (**E**) MGA without ACh induction, (**F**) MGA with ACh induction, (**G**) MGA with ACh induction and pretreated with L-NAME, (**H**) MGA with ACh induction and pretreated with ABH. Bar: 20 µm. Fluorescence reflects NO production, which was more intense in cells induced with acetylcholine than in cells without acetylcholine. MGA-treated cells had lower fluorescence, indicating lower NO production even with acetylcholine induction (**F**); however, when pretreated with ABH (**H**), fluorescence induced by ACh was intensified and NO was restored to a level higher than ACh-induced, MG-treated cells (**F**). L-NAME inhibitor abolished ACh-induced fluorescence, reflecting inhibition of eNOS activity and NO production. A quantification of DAF fluorescence intensity in the different treatment conditions is demonstrated in (**I**). Values are expressed as percentage of BSA (control); analyzed images were obtained from 3 independent experiments. * *p* < 0.05. ACh, acetylcholine (1 µM); L-NAME, N (G)-nitro-L-arginine methyl ester (1 µM); ABH, boronic acids 2(S)-amino-6-boronohexanoic acid (1 mM).

Intracellular NO generation was assessed in MAECs utilizing the DAF-2DA marker. Subsets of cells were treated with BSA as a control (100 μM, 24 h) (Figure 3A); the addition of ACh (1 μM) to BSA-treated cells induced an increase in the DAF-2DA fluorescence, reflecting NO generation (Figure 3B) compared with no ACh in Figure 3A. Pretreatment with L-NAME (1 mM) reduced ACh-induced NO production (Figure 3C), while ACh-induced NO production increased with pretreatment with the arginase inhibitor ABH (1 mM) (Figure 3D). Another subset of cells were pretreated with MGA (100 μM, 24 h), which demonstrated nearly undetectable fluorescence without ACh stimulation (Figure 3E); NO production increased slightly after the addition of ACh in MGA-treated cells (Figure 3F), whereas the L-NAME inhibitor blunted NO production in ACh-stimulated, MGA-treated cells (Figure 3G). Interestingly, pretreatment with the ABH inhibitor rescued NO production to close to the control ACh-stimulated cells (Figure 3H). A quantification of DAF fluorescence intensity in the different treatment conditions is depicted in Figure 3I). It is noteworthy that ABH restoration of ACh-induced NO production, indicated by increased fluorescence intensity in BSA treatment, was reversed by L-NAME inhibition to a level less than when ABH was not used, while eNOS was inhibited by L-NAME, confirming that this effect of ABH is rather due to the inhibition of arginase enzyme and not the stimulation of eNOS (Figure 3I).

### 3.4. Vascular Function

**Figure 4:**
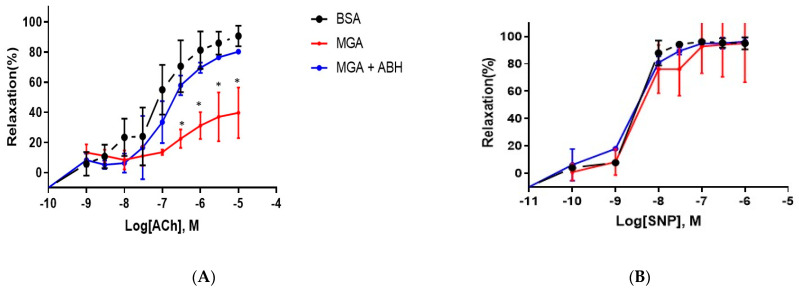
Dose-response relaxation curves for (**A**) endothelium-dependent vasorelaxant acetylcholine (ACh) in phenylephrine (1 μM)-preconstricted aortas from mice; (**B**) endothelium-independent vasorelaxant sodium nitroprusside (SNP) in phenylephrine (1 μM)-preconstricted aortas from mice. Dashed black line indicates responses in control conditions (BSA, 100 μM, 24 h); solid red line indicates responses in MGA-pretreated aortas (100 μM, 24 h); solid blue line indicates responses in MGA-treated aortas pretreated with ABH (1 mM, 24 h). n = 3 in each group; * *p* < 0.05 MGA vs. control or MGA + ABH.

To determine the effect of MGA on endothelial function in vivo, we performed vascular studies using aortas isolated from C57BL/6J healthy mice. We examined vasorelaxation responses to the endothelium-dependent vasodilator ACh and the endothelium-independent vasodilator SNP (Figure 4). Pretreatment of isolated aortas with MGA (100 μM, 24 h) induced an impairment of vasorelaxation response to ACh (maximum relaxation of 39.7 ± 5.7% vs. 90.7 ± 1.7% in control condition, *p* < 0.05, n = 3–5 independent experiments), as shown in Figure 4A. ABH largely prevented MGA-impaired vasorelaxation with a maximum relaxation of 80.4 ± 5.3%, *p* < 0.05, n = 3–5 independent experiments. Thus, blocking arginase activity reversed MGA-induced impairment. Aortic relaxation responses to SNP were not different between control, MGA-treated rings or ABH- and MGA-treated rings, as demonstrated in Figure 4B. ABH pretreatment of control rings (BSA) did not affect vasorelaxation responses to either ACh or SNP (data not shown).

## 4. Discussion

**Figure 5:**
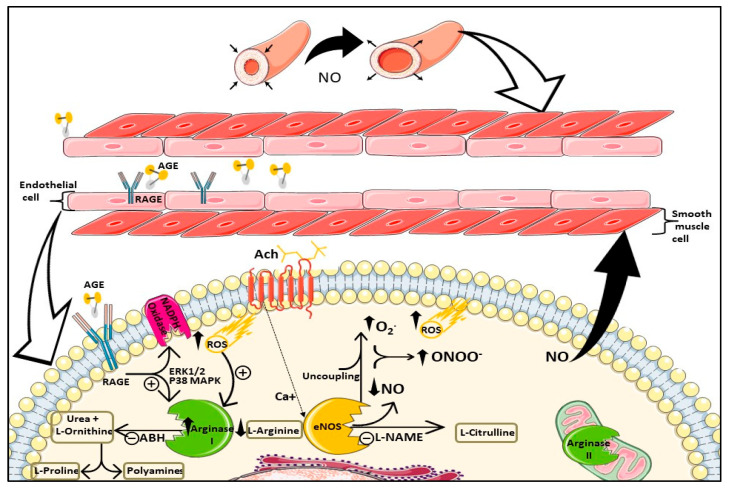
A schematic diagram of AGE/RAGE interaction with arginase enzyme and its effects on endothelial cells and vascular function. Acetylcholine (ACh) stimulates eNOS to produce NO, which is released from endothelial cells to smooth muscle cells, inducing vasorelaxation. Circulating AGE binding to RAGE activates NADPH oxidase, producing ROS, and stimulates ERK1/2 and P38 MAPK, which induce activity/expression of arginase I enzyme. Upregulation of arginase I limits both arginine and NO production by eNOS. Limited arginine leads to uncoupling of eNOS, which further limits NO production and produces superoxide (O_2_^.^) that reacts with NO, generating peroxinitrite (ONOO-) and further reducing NO. Arginase activation produces urea and L-ornithine that is used to produce L-proline and polyamines involved in collagen formation and proliferation, respectively. Arginase II is expressed in mitochondria and may be regulated by AGE by a different mechanism not involving its expression. Abbreviations: AGE: advanced glycation end products, RAGE: receptor for advanced glycation end products, ROS: reactive oxygen species, ABH: arginase inhibitor, L-NAME: eNOS inhibitor. Some components of the figure were drawn by using pictures from Servier Medical Art. Servier Medical Art by Servier is licensed under a Creative Commons Attribution 3.0 Unported License (https://creativecommons.org/licenses/by/3.0/) (accessed on 3 November 2022).

This study demonstrates for the first time that advanced glycated end products represented by methylglyoxal-modified albumin stimulates arginase enzyme activity in an ERK1/2 MEKK and p38 MAPK-dependent pathway, as summarized in Figure 5. Increased activity is mainly due to increased arginase I expression, as shown in our study. Our findings support previous reports showing that constitutive levels of arginase activity in endothelial cells limit NO synthesis and NO-dependent vasodilatory function [6,7,8]. In hyperglycemic conditions, both AGEs and arginase have been individually linked to various diabetic complications, including vascular dysfunction; however, in the literature, there is a lack of studies investigating if there is a direct influence of AGEs on arginase regulation. Previously, AGE-modified albumin was shown to have suppressive effects on NOS-3 activity and expression in HUVECs, an effect that if combined with upregulation of arginase, would aggravate limited NO bioavailability and VD [41].

Intracellular detection of NO in cultured endothelial cells in our study showed that MGA-induced increased activity and expression of arginase was accompanied by a reduction in NO bioavailability. Furthermore, we show that MGA treatment of aortic rings impaired endothelial-dependent vasodilation in response to ACh, which was reversed by the arginase inhibition (ABH) without affecting SNP-induced (endothelial-independent) vasorelaxation, suggesting a role for endothelial arginase enzyme in MGA-induced vascular impairment. In accordance with these findings, aortic rings treated with AGE demonstrated blunted endothelial-dependent vasorelaxation. These findings were consistent with a previous report by Watson’s group in which AGE treatment of rat aortic rings impaired endothelial-dependent vasodilation that was blocked by inhibition arginase, NADPH oxidase, and superoxide [42]. We showed no alteration of endothelial-independent relaxation; however, they showed increased endothelial independent vasodilation by AGE [42]. Furthermore, they reported increased arginase and NADPH oxidase mRNA expression with MGA treatment, which may not be necessarily predictive for protein expression. On the contrary, our study showed an increase in both activity and protein expression of arginase enzyme upon MGA treatment. Similar to our findings, coronary arteries obtained from diabetic patients had increased protein levels of arginase I and showed a better vasodilation response to ACh in the presence of the arginase inhibitor [24]. Moreover, we provide evidence of reduced NO production using the intracellular marker DAF-2DA, whereas arginase inhibition with ABH restored ACh-induced NO production in cultured endothelial cells treated with MGA, which explains our vascular function findings.

In concordance with our findings that arginase I expression was preferentially increased by AGEs, arginase knockout mice models suggested that arginase I is crucial in diabetes-induced vascular dysfunction. One study showed that streptozotocin-induced diabetic knockout mice lacking the arginase II with partial deletion of arginase I exhibited better endothelial-dependent vasodilation and less arginase activity compared with diabetic wild-type and knockout mice lacking the AII isoform alone [18].

A growing body of evidence indicates that AGE receptor (RAGE) engagement by its ligands including AGE stimulate NADPH oxidase, reactive oxygen species (ROS) production, ERK1/2, P38 MAP-kinase, NFκB activation, and gene transcription, culminating in microvasculature alterations manifested in diabetes [43,44,45]. Arginase expression/activity has been extensively shown to be stimulated by a wide range of stimuli involving oxidative stress when administered to cultured endothelial cells, including high glucose [15], oxidized low-density lipoprotein (LDL) [12], H_2_O_2_ [5,46,47], peroxynitrite [9], and endotoxins [10]. Additionally, in vivo studies revealed that conditions well known to be associated with elevated oxidative stress have elevated endothelial arginase expression, such as ischemia–reperfusion [48] and ageing [49].

Moreover, AGEs via RAGE receptors as well as arginase-induced eNOS uncoupling may lead to ROS formation, including superoxide (O2-) ion, which further combines with NO to form the potent oxidant peroxynitrite, limiting NO bioavailability and aggravating the oxidative injury to endothelial cells [50]. Taken together, AGE-induced arginase upregulation might result from AGE-stimulated ROS formation and might contribute to AGE-induced ROS loop at the same time.

Arginase activation was linked to protein kinase C (PKC), Rho-associated protein kinase (ROCK), and the mitogen-activated protein kinase (MAPK) pathways [9,51,52]. Post-translational modifications such as S-nitrosylation of arginase I via inducible NOS2 have been identified in age-related endothelial dysfunction [53]. In addition, the physiologic modulation of the glutathione/glutathione disulfide ratio has been suggested to play a role in the control of arginase I activity in pathological conditions of increased oxidative stress [13].

Although we show no changes in protein expression of arginase II, it may contribute to increased arginase activity by other activating mechanisms. Pandey et al. demonstrated a mechanism for rapid arginase II increased activity via translocation from mitochondria to cytoplasm in response to oxidized LDL interaction with LOX1 receptor causing NO dysregulation and vascular dysfunction [54]. AGEs were reported to bind the LOX1 receptor, presenting a compelling mechanism for arginase II contribution to increased arginase activity that requires further investigation [55,56].

In concordance to the previous evidence that hyperglycemia-induced dysregulation of NO and increased generation of ROS as well as endothelial dysfunction are maintained even after the restoration of normoglycemia, known as hyperglycemic memory phenomenon, we observed from previous studies that the degree of endothelial function improvement achieved by arginase inhibition was independent of glucose control, which can be partly explained by the role of the AGEs/RAGE axis involved in this phenomenon [57,58,59].

These intriguing observations highlight the role of AGE in arginase regulation of NO and oxidative stress, which may present a putative therapeutic target to maintain cardiovascular integrity and function in diabetes.

## 5. Conclusions

Based on our findings, we conclude that AGEs affect VD by upregulating arginase activity and expression, thus limiting NO bioavailability in endothelial cells. This study emphasizes the importance of further investigating the interaction between AGEs and arginase enzymes, particularly in diabetes.

## Data Availability

All data presented in this study are available upon request from the corresponding author. Data are contained within the article and Supplementary Materials.

## Supplementary Materials

The following supporting information can be downloaded at: https://www.mdpi.com/article/10.3390/cells12050795/s1, Figure S1: The graph represents concentrations of ABH for arginase enzyme activity inhibition in MAEC cells. * *p* < 0.01 control vs. ABH. Concentration of 100 μM and above significantly inhibited arginase activity in MAEC cells. Values are expressed as means ± SE from 5 independent experiments carried out in triplicates.
