# Supplementary material for: Methylglyoxal-Modified Albumin Effects on Endothelial Arginase Enzyme and Vascular Function
Source: Cells. 2023 Mar 3;12(5):795. doi: 10.3390/cells12050795 (PMC10001288; doi:10.3390/cells12050795)
Supplement: Supplementary file 1 [file cells-12-00795-s001.zip › cells-2143843-supplementary.pdf]

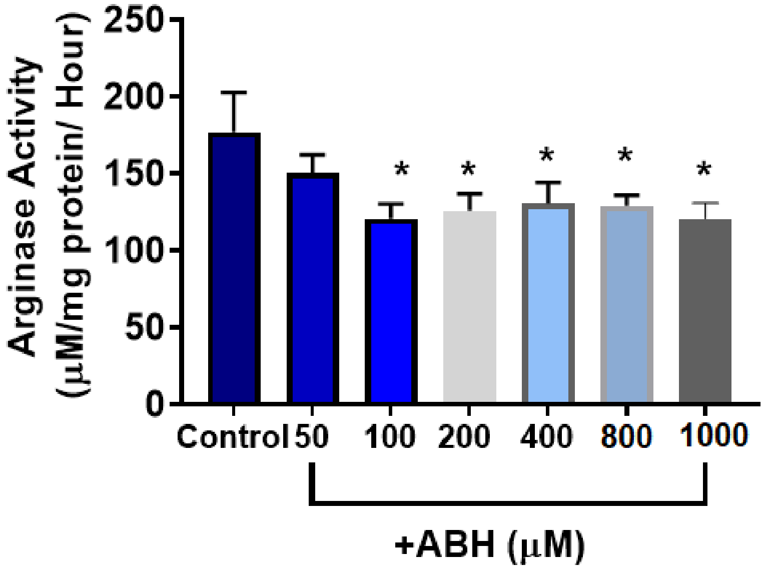

Figure S1 : The graph represents concentrations of ABH for arginase enzyme activity inhibition in MAEC cells. *$p < 0.01$ control vs. ABH. Concentration of 100 µM and above significantly inhibited arginase activity in MAEC cells. Values are expressed as means± SE from 5 independent experiments carried out in triplicates.
